# Association Between Asthma and Gout: A Longitudinal Follow-Up Study Using a National Health Screening Cohort

**DOI:** 10.3390/biomedicines13040819

**Published:** 2025-03-28

**Authors:** Heejin Kim, Tae Jun Kim, Mi Jung Kwon, Jee Hye Wee, Sung Kwang Hong, Hyo Geun Choi, Joong Seob Lee

**Affiliations:** 1 Department of Otorhinolaryngology-Head and Neck Surgery, Hallym University Sacred Heart Hospital, Hallym University College of Medicine, Anyang 14068, Republic of Korea; 2 Department of Medicine, Samsung Medical Center, Sungkyunkwan University School of Medicine, Seoul 06351, Republic of Korea; 3 Department of Pathology, Hallym University Sacred Heart Hospital, Hallym University College of Medicine, Anyang 14068, Republic of Korea; 4 Suseo Seoul ENT Clinics, Seoul 06349, Republic of Korea

**Keywords:** gout, asthma, cohort study

## Abstract

**Background**: Previous reports suggest a connection between gout and asthma; however, additional research is required to clarify this link. This study explores the relationship between gout and asthma using data from the Korean National Health Insurance Service-Health Screening Cohort. **Methods**: Participants were selected according to medical claim codes, and individuals diagnosed with gout were paired with control subjects at a 1:4 ratio. Variables, including demographic characteristics, health-related information, and medical history, were incorporated into the analysis. The incidence rates and hazard ratios of asthma were examined. Additionally, a comprehensive analysis was conducted to investigate the relationship between gout and asthma exacerbation. **Results**: Among the 514,866 participants, 19,830 patients with gout and 79,320 matched controls were analyzed. After adjusting for variables, the gout group demonstrated a significantly higher risk of asthma compared with the control group (adjusted hazard ratio [HR], 1.11, *p* < 0.001). Compared with the control group, the Kaplan–Meier method and log-rank test revealed a statistically significant increase in the cumulative incidence of asthma in the gout group over a 17-year period. However, the gout group did not show a significantly higher hazard ratio for asthma exacerbation. **Conclusions**: This study demonstrated that gout was associated with an increased risk of asthma. Additional analysis showed that gout was not correlated with asthma exacerbation. Further research is needed to fully elucidate the association between gout and asthma.

## 1. Introduction

Gout is a prevalent type of arthritis resulting from the accumulation of monosodium urate crystals in the connective tissues of joints, with a particular predilection for the foot [1]. This condition is marked by repeated occurrences of severe joint inflammation, commonly referred to as gout flares. Recent studies on the prevalence of gout exhibit considerable variability depending on the population examined and methodologies applied, with a reported prevalence ranging from less than 1% to 6.8% [2]. Individuals with gout often present with multiple comorbid conditions, such as high blood pressure, chronic renal disease, cardiovascular disorders, overweightness, diabetes, and dyslipidemia, all of which contribute substantially to the burden on public health [3]. Due to the high recurrence rate, a significant number of patients with gout experience a decline in health-related quality of life and an increased financial burden, which ultimately leads to poor gout control [4,5]. Furthermore, patients with gout who are also diagnosed with cardiovascular disease exhibit a higher mortality rate, irrespective of age, sex, or comorbid conditions [6].

Asthma is a common chronic inflammatory disorder characterized by restricted airflow, increased airway reactivity, and structural remodeling of the respiratory passages [7]. Patients with asthma experience a range of symptoms, including coughing, chest tightness, wheezing, and shortness of breath [8]. The prevalence of asthma was reported to be approximately 8.3% in a prior study that analyzed data from the National Health and Nutrition Examination Survey (NHANES) [9]. Asthma is recognized as a major public health concern, frequently necessitating emergency medical intervention, including hospitalizations, and contributing to a substantial number of absences from school and work. Notably, uncontrolled asthma imposes considerable restrictions on patients’ physical activity, social interactions, and professional or academic performance [10,11]. Moreover, the economic burden associated with asthma is very high [12].

Since gout and asthma impose significant burdens on both patients and society, their potential association has been the subject of ongoing research [13,14,15]. Even in the recent literature, elevated serum uric acid levels have been reported as an independent risk factor for the development of asthma in men [14]. The association between elevated uric acid levels and the development of asthma may be attributed to the pro-inflammatory effect of uric acid in the respiratory mucosa. For example, uric acid crystals can promote Th2 cell immunity [16], which is linked to airway eosinophilia and bronchial hyperreactivity. However, the precise mechanisms underlying gout and asthma have not yet been fully elucidated. In addition, previous studies were either of a cross-sectional design, which posed challenges in establishing causality [15], or involved relatively small sample sizes, limiting the ability to accurately assess the relationship between gout and asthma [13,17].

In this study, we conducted a longitudinal follow-up analysis using a population-based national cohort to examine the association between gout and asthma. To enhance the accuracy of the analysis, detailed adjustments were made for sociodemographic variables, lifestyle characteristics, laboratory findings, and medical history. Furthermore, supplementary analyses were performed to investigate the relationship between gout and asthma exacerbation.

## 2. Materials and Methods

### 2.1. Ethics

This study was approved by the Ethics Committee of Hallym University (2019-10-023). The Institutional Review Board granted a waiver for written informed consent. All analyses were conducted in accordance with the guidelines and regulations set forth by the Hallym University Ethics Committee. A comprehensive description of the Korean National Health Insurance Service-Health Screening (NHIS-HealS) Cohort data (2002–2003, with follow-up until 2019) is provided elsewhere [18]. The NHIS-HealS Cohort data are publicly available, but access requires permission through the NHIS (National Health Insurance Service) database. Researchers must apply for access and follow the appropriate procedures to use the data.

### 2.2. Exposure (Gout)

Gout was defined as participants who visited a medical facility with the diagnosis of gout (M10, ICD-10 code) on two or more occasions. These methods were adapted from a previous study [19].

### 2.3. Outcome (Asthma)

Participants diagnosed with asthma (J45, ICD-10 code) or status asthmaticus (J46, ICD-10 code) from 2002 through 2013 were included in this study. From this group, we selected participants who had been diagnosed with asthma by a physician on more than two occasions and who had been treated with asthma-related medications. These medications included inhaled corticosteroids (ICSs) or ICSs combined with long-acting β2-agonists (LABAs), oral leukotriene antagonists (LTRAs), short-acting β2-agonists (SABAs), systemic LABAs, xanthine derivatives, or systemic corticosteroids [20]. Asthma exacerbation was characterized by instances where asthma patients required emergency medical attention or hospital admissions beyond their initial visit for asthma. Additionally, individuals prescribed corticosteroids at a dosage of ≥20 mg per day for at least three consecutive days within a two-week period were also classified as experiencing asthma exacerbation [21].

### 2.4. Participants Selection

**Figure 1:**
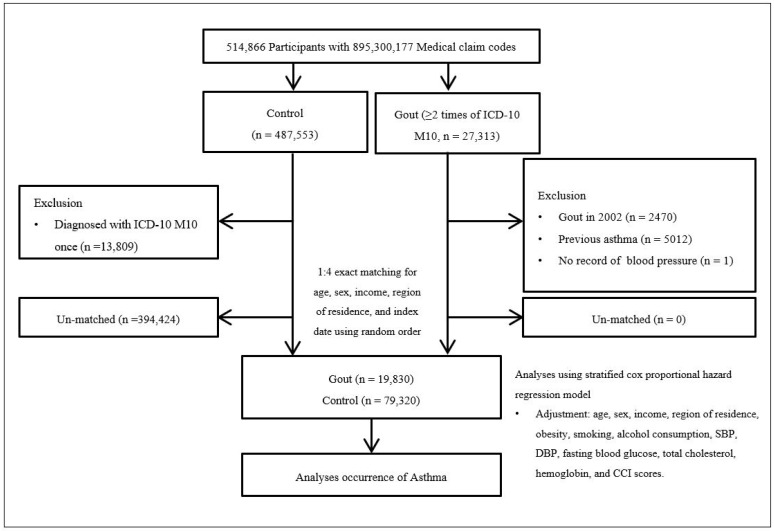
A schematic representation of the participant selection process. From a total cohort of 514,866 individuals, 19,830 patients with gout were matched with 79,320 control participants based on age, sex, income, and region of residence. Abbreviation: ICD-10—International Classification of Diseases-10; CCI—Charlson Comorbidity Index; SBP—systolic blood pressure; DBP—diastolic blood pressure.

A detailed description of the Korean National Health Insurance Service-Health Screening Cohort data is provided elsewhere [18]. Participants with gout were selected from a pool of 514,866 individuals with 895,300,177 medical claim codes from 2002 through 2019 (n = 27,313). The control group included participants who were not diagnosed with gout during this period (n = 487,553). To identify participants diagnosed with gout for the first time, those diagnosed in 2002 were excluded (washout period, n = 2470). Control participants were also excluded if they had a single diagnosis of gout (M10, ICD-10 code) (n = 13,809). Both gout and control groups excluded participants with a history of asthma prior to the index date, resulting in the exclusion of 5012 participants with gout. Additionally, participants with gout without a record of blood pressure were excluded (n = 1). Participants with gout were matched 1:4 with control participants based on propensity score matching (PSM). The propensity scores were estimated using logistic regression, adjusting for age, sex, income, and region of residence. A caliper width of 0.2 was applied to ensure optimal matching between gout and control groups. To prevent selection bias, control participants were sorted randomly and selected sequentially. Control participants who died before the index date were also excluded. During the matching procedure, 394,424 control participants were excluded. Ultimately, 19,830 participants with gout were matched with 79,320 control participants (Figure 1).

### 2.5. Covariates

Participants were stratified into age groups spanning 5-year intervals starting from 40–44 years up to 85 years and older, resulting in a total of 10 distinct age categories. Income levels were segmented into five classes, ranging from class 1 (lowest income) to class 5 (highest income). The region of residence was dichotomized into urban areas (including Seoul, Busan, Daegu, Incheon, Gwangju, Daejeon, and Ulsan) and rural areas (comprising Gyeonggi, Gangwon, Chungcheongbuk, Chungcheongnam, Jellabuk, Jellanam, Gyeongsanbuk, Gyeongsangnam, and Jeju).

Smoking status was classified into three categories: nonsmokers, former smokers, and current smokers. Alcohol consumption frequency was divided into two groups: less than once a week and once or more per week. Obesity was assessed using the body mass index (BMI, kg/m^2^), which was categorized according to the Asia–Pacific criteria established by the Western Pacific Regional Office (WPRO) in 2000 [22]: underweight (BMI < 18.5), normal range (BMI ≥ 18.5 to <23), pre-obese (BMI ≥ 23 to <25), class I obesity (BMI ≥ 25 to <30), and class II obesity (BMI ≥ 30).

Assessments included systolic and diastolic blood pressure (mmHg), fasting plasma glucose (mg/dL), and total serum cholesterol (mg/dL). The Charlson Comorbidity Index (CCI) was used to quantify disease burden, incorporating 17 comorbid conditions. Each participant was assigned a score reflecting both the quantity and severity of these conditions, with CCI values spanning from 0 (no comorbidities) to 29 (multiple comorbidities) [23].

### 2.6. Statistical Analyses

Standardized difference was employed to compare the general characteristic rates between the gout and the control groups. To evaluate the hazard ratios (HRs) and 95% confidence intervals (CIs) of gout for asthma, stratified Cox proportional hazard models were utilized. For patients with asthma, unstratified Cox proportional hazard models were also employed to determine the HRs and 95% CIs for asthma exacerbation due to gout. Both crude (unadjusted) and adjusted (for obesity, smoking, alcohol consumption, systolic blood pressure, diastolic blood pressure, fasting blood glucose, total cholesterol, and CCI scores) models were used in these analyses, with 95% CIs calculated. The analyses were stratified by age, sex, income, and region of residence. Additionally, Kaplan–Meier curves and the log-rank test were applied.

For subgroup analyses using the stratified Cox proportional hazards model, participants were divided based on age (<60 years and ≥60 years), sex, obesity (underweight, normal weight, overweight, obese I, and obese II), and fasting blood glucose levels (<100 mg/dL and ≥100 mg/dL).

Additional subgroup analyses were conducted using the unstratified Cox proportional hazards model, dividing participants by income (class 1 [lowest], class 2, class 3, class 4, and class 5 [highest]), region of residence (urban and rural), smoking status (nonsmoker, past smoker, and current smoker), alcohol consumption frequency (<1 time per week and ≥1 time per week), systolic blood pressure (<120 mmHg, ≥120 mmHg and <140 mmHg, ≥140 mmHg), diastolic blood pressure (<80 mmHg, ≥80 mmHg and <90 mmHg, ≥90 mmHg), total cholesterol levels (<200 mg/dL, ≥200 mg/dL and <240 mg/dL, ≥240 mg/dL), and CCI scores (0, 1, and ≥2).

All analyses were two-tailed, with significance set at *p* values less than 0.05. Statistical analyses were conducted using SAS version 9.4 (SAS Institute Inc., Cary, NC, USA).

## 3. Results

In this study, a total of 19,830 individuals diagnosed with gout were included, along with 79,320 matched controls based on pertinent criteria. The demographic and clinical characteristics of the study participants are presented in Table 1. Except for obesity, most variables exhibited standardized differences not exceeding 0.2, indicating similarity between the gout and the control groups. The standardized difference for obesity was 0.27, reflecting a slight disparity in the distribution of obesity between the two groups.

Asthma occurred in 1898 individuals (9.57%) in the gout group and 6668 individuals (8.41%) in the control group. The incidence rate of asthma was 12.50 (per 1000 person-years) in the gout group and 10.90 (per 1000 person-years) in the control group. After adjusting for covariates, the HR for asthma was 1.11 (95% CI: 1.05–1.17, *p* < 0.001) (Table 2).

**Figure 2:**
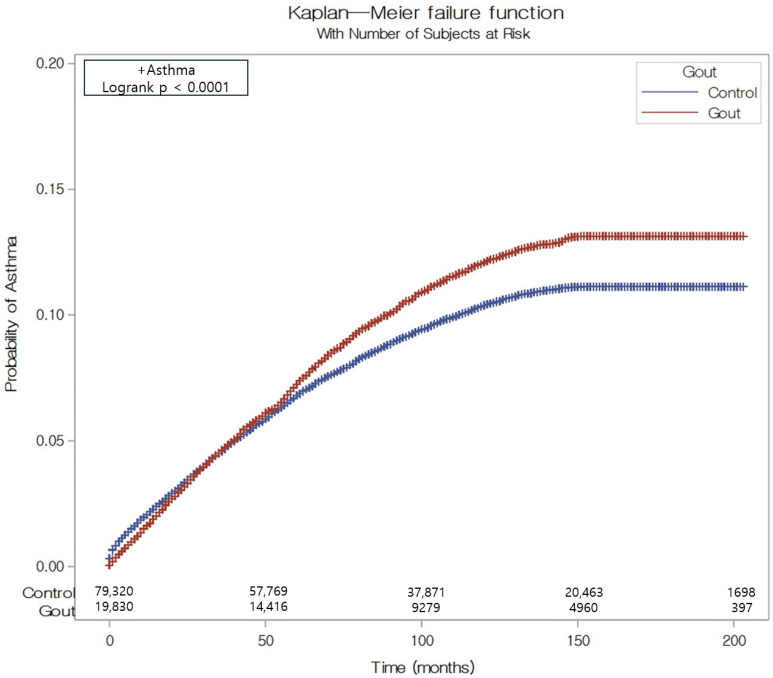
The Kaplan–Meier probability of the incidence of asthma in participants with gout and control participants.

The subgroup analysis exposed variations in the risk of asthma among participants with gout. After adjustment using the overlap weighting method, the gout group demonstrated a markedly elevated HR for asthma in most subgroups, particularly among those with low income, residents of rural areas, and individuals with normal or overweight status (Table 2). The Kaplan–Meier approach combined with a log-rank test revealed a statistically significant increase in the cumulative incidence of asthma in the gout group over the study period (Figure 2).

**Figure 3:**
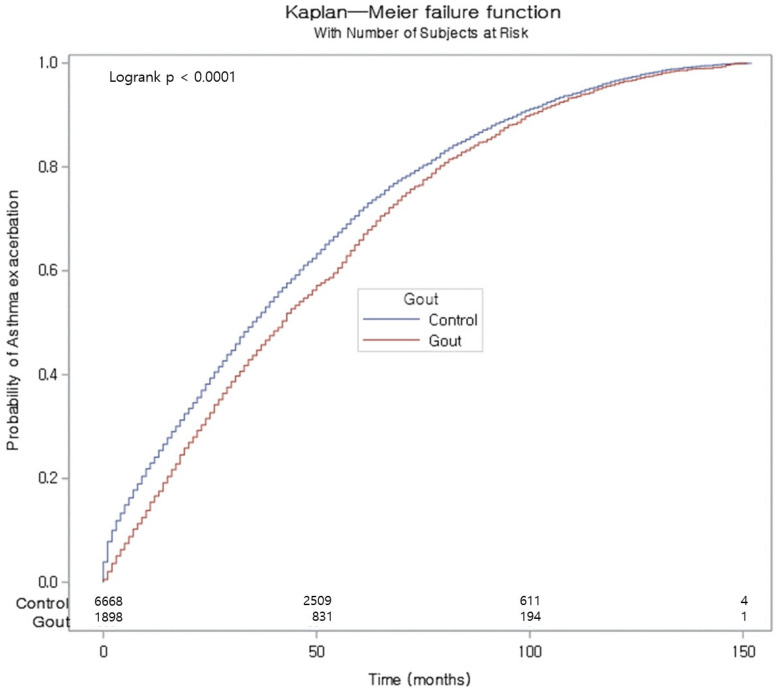
Kaplan–Meier probability of the incidence of asthma exacerbation in patients with gout and the control participants.

In a supplementary analysis, we assessed HR for asthma exacerbation. The gout group did not demonstrate a markedly increased HR for asthma exacerbation (Table 3). However, in the underweight group, the gout demonstrated a significantly increased HR for asthma exacerbation. The Kaplan–Meier approach combined with a log-rank test demonstrated a statistically significant increase in the cumulative incidence of asthma exacerbation in the control group over the study period (Figure 3).

## 4. Discussion

This study showed a significant association between gout and asthma. The results of this study suggest that individuals with gout have a more elevated risk of developing asthma than healthy individuals. This connection remained significant even after accounting for demographic variables and existing health conditions.

A few previous studies have proposed an association between gout and the development of asthma [13,14,15]. Similar to our findings, a cross-sectional study utilizing data from a national database reported an adjusted OR of 1.15 (95% CI 1.13–1.17) for the association between all types of asthma and gout [15]. However, these studies relied on cross-sectional designs, which limited the ability to establish a temporal relationship between gout and asthma, or they were conducted with relatively small sample sizes.

Some possible pathophysiologic mechanisms could explain the association between gout and asthma. Firstly, inflammation associated with uric acid may play a role in the development of asthma. Uric acid is recognized as a highly efficient antioxidant in the upper airway, actively neutralizing peroxynitrite and eliminating reactive oxygen species (ROS) [24]. However, it also exhibits pro-inflammatory properties within endothelial tissue [25]. A potential inflammatory mechanism triggered by uric acid may involve the profibrotic factor endothelin-1 [26]. Uric acid induces the upregulation of endothelin-1 expression [27], and inflammatory mediators, such as endothelin-1, IL-6, and IL-8, have been shown to promote mucus secretion, airway swelling, and bronchial hyperreactivity [28]. Therefore, elevated uric acid levels in patients with gout may play a significant role in triggering inflammatory responses in the airway, potentially contributing to the onset or exacerbation of asthma.

Secondly, the presence of inflammasome may contribute to the development of asthma. Notably, the nucleotide-binding oligomerization domain-like receptor family pyrin domain containing 3 (NLRP3) inflammasome has attracted significant attention due to its involvement in inflammatory disorders [29]. The NLRP3 inflammasome is a protein complex that plays a critical role in the innate immune defense mechanism within the respiratory tract. Exposure to inhaled irritants or allergens can trigger the activation of the NLRP3 inflammasome, potentially resulting in the aggravation of asthma symptoms and pulmonary inflammation [30]. Monosodium urate (MSU) crystals, which accumulate in the joints of patients with gout, serve as activators of the NLRP3 inflammasome [31]. This activation induces the release of pro-inflammatory cytokines, including IL-1β and IL-18, driving the characteristic symptoms of gout attacks [31].

In the subgroup analysis, a positive correlation between gout and asthma was observed across the entire participants and specific subgroups, such as individuals with low incomes, those residing in rural areas, and participants classified as normo-overweight demonstrated statistically significant associations (Table 2). The association between low socioeconomic status and the development of gout or asthma has been reported in previous studies [32,33]. Individuals with higher income levels or those residing in urban areas tend to have better access to healthcare systems. Our findings suggest that socioeconomic status may contribute to the increased incidence of asthma among patients with gout. However, previous studies have also indicated that urban living itself may contribute to a higher risk of asthma due to environmental factors such as air pollution and increased exposure to allergens [34,35]. Therefore, the relationship between socioeconomic status, urbanization, and asthma is complex, and all factors should be considered when interpreting our findings.

Although a non-significant association between gout and asthma exacerbation was observed across the entire participants, the underweight group demonstrated a statistically significant association (Table 3). Obesity is generally considered a major risk factor for asthma exacerbation [36]. However, a recent study reported a higher prevalence of asthma among underweight individuals [37], suggesting that BMI plays a complex role in asthma risk. In addition, another study found that underweight patients with asthma demonstrated lower scores on the asthma control test (ACT) compared with their normal-weight counterparts [38]. Individuals in this population often have lower nutritional reserves and muscle mass than those with normal weight, which might contribute to respiratory muscle weakness and worsening asthma symptoms. Despite these findings, the influence of hyperuricemia on frequent asthma exacerbation in underweight patients with gout remains unclear. Further complementary studies are warranted to substantiate this association.

In our Kaplan–Meier analysis, the gout group demonstrated a significantly increased cumulative incidence of asthma over the study period compared with the control group (Figure 2). Meanwhile, our results exhibited a significantly increased cumulative incidence of asthma exacerbation over the study period in the control group (Figure 3). The possible explanations for the significant increased cumulative incidence of asthma in patients with gout have been discussed in the previous section, including uric acid-related inflammation and the presence of NLRP3 inflammasome. However, explaining the significantly increased cumulative incidence of asthma exacerbation in the control group remains challenging. Several potential explanations can be considered for this finding. Firstly, this phenomenon can be explained by anti-inflammatory and antioxidant properties of uric acid. Similar to our findings, Luo et al. reported in their national database study that gout acts as a protective factor for asthma exacerbation [15]. In their study, the odds ratio (OR) of asthma exacerbation in patients with gout was 0.87 (95% CI 0.83–0.91, *p* < 0.001). This finding may be attributable to the distinct biochemical properties of uric acid, which exhibit both pro-inflammatory and antioxidant characteristics, as suggested in their report. Consequently, the presence of uric acid may provide patients with gout with greater protection against asthma exacerbation compared with the control group. Secondly, asthma exacerbation may occur more frequently in the control group due to the influence of medication. Patients with gout are frequently prescribed uric acid-lowering medications, such as allopurinol and probenecid. Therefore, it is possible that individuals classified in the gout group may have maintained lower uric acid levels compared with the control group. For a more accurate evaluation, further studies are needed to explore the correlation between serum uric acid levels, medication-induced changes in uric acid levels, and asthma exacerbation.

This study has several limitations. First, the participant selection was based on medical claim codes and diagnostic codes derived from the national database. While diagnostic codes are generally indicative of a gout diagnosis, the laboratory data would offer a higher degree of diagnostic accuracy. Consequently, this study does not account for the severity of gout. Second, the use of various medications for treating gout and asthma may influence the results. Specifically, drugs used for joint pains, such as nonsteroidal anti-inflammatory drugs (NSAIDs) or corticosteroids, may impact asthma. Third, dietary factors were not considered in this study. Given that diet plays a crucial role in both gout and asthma, the lack of dietary information limits our ability to assess its potential impact on the observed association. Future studies incorporating detailed dietary data may provide a more comprehensive understanding of this relationship. Fourth, while we employed a Cox proportional hazards model to estimate the association between gout and asthma, the proportional hazards assumption may not be fully satisfied. As shown in Figure 2, asthma incidence rates were similar between the gout and control cohorts for the first 4–5 years, with divergence occurring thereafter. This pattern suggests a potential time-dependent effect, which could influence the estimated hazard ratio. To address this, future studies employing time-dependent Cox models or alternative statistical approaches could provide a more refined understanding of the temporal relationship between gout and asthma.

Even with these limitations, this study contains several notable strengths. First, a comprehensive analysis was conducted using a large dataset drawn from a national cohort, enhancing this study’s reliability. To the best of our knowledge, this represents the most extensive investigation to date exploring the relationship between gout and asthma. Second, potential sources of bias were effectively minimized. Rigorous selection criteria for both gout and controls helped mitigate selection bias. Furthermore, participants were carefully matched based on relevant baseline characteristics, and adjustments were made to account for a wide range of potential confounders. These confounders included factors associated with both gout and asthma, addressing the potential interrelationship between these conditions.

## 5. Conclusions

This study demonstrated that gout was related to increased asthma risk. This effect was enhanced in a participant with low income, rural resident, and normo-overweight. Additional analysis demonstrated that gout was not correlated with asthma exacerbation. Further research should be required to elucidate the association between gout and asthma.

## Figures and Tables

**Table 1:** Baseline characteristics of the participants.

Characteristics		Total Participants	
	Gout	Control	SD
Age (years old) (n, %)			0.00
40–44	562 (2.83)	2248 (2.83)	
45–49	1937 (9.77)	7748 (9.77)	
50–54	3189 (16.08)	12,756 (16.08)	
55–59	4064 (20.49)	16,256 (20.49)	
60–64	3422 (17.26)	13,688 (17.26)	
65–69	2757 (13.90)	11,028 (13.90)	
70–74	1995 (10.06)	7980 (10.06)	
75–79	1192 (6.01)	4768 (6.01)	
80–84	553 (2.79)	2212 (2.79)	
85+	159 (0.80)	636 (0.80)	
Sex (n, %)			0.00
Male	16,169 (81.54)	64,676 (81.54)	
Female	3661 (18.46)	14,644 (18.46)	
Income (n, %)			0.00
1 (lowest)	2814 (14.19)	11,256 (14.19)	
2	2425 (12.23)	9700 (12.23)	
3	3017 (15.21)	12,068 (15.21)	
4	4180 (21.08)	16,720 (21.08)	
5 (highest)	7394 (37.29)	29,576 (37.29)	
Residential area (n, %)			0.00
Urban	8482 (42.77)	33,928 (42.77)	
Rural	11,348 (57.23)	45,392 (57.23)	
Obesity (n, %)			0.27
Underweight	258 (1.30)	1856 (2.34)	
Normal	4953 (24.98)	27,572 (34.76)	
Overweight	5459 (27.53)	22,510 (28.38)	
Obese I	8311 (41.91)	25,480 (32.12)	
Obese II	849 (4.28)	1902 (2.40)	
Smoking status (n, %)			0.03
Non-smoker	10,563 (53.27)	42,084 (53.06)	
Past smoker	2852 (14.38)	10,343 (13.04)	
Current smoker	6415 (32.35)	26,893 (33.90)	
Alcohol consumption (n, %)			0.11
<1 time a week	11,166 (56.31)	49,094 (61.89)	
≥1 time a week	8664 (43.69)	30,226 (38.11)	
SBP (n, %)			0.16
<120 mmHg	4680 (23.60)	22,430 (28.28)	
120–139 mmHg	9779 (49.31)	39,685 (50.03)	
≥140 mmHg	5371 (27.09)	17,205 (21.69)	
DBP (n, %)			0.14
<80 mm Hg	7736 (39.01)	35,233 (44.42)	
80–89 mmHg	7545 (38.05)	29,407 (37.07)	
≥90 mmHg	4549 (22.94)	14,680 (18.51)	
Fasting blood glucose (n, %)			0.01
<100 mg/dL	11,172 (56.34)	46,197 (58.24)	
100–125 mg/dL	6631 (33.44)	24,412 (30.78)	
≥126 mg/dL	2027 (10.22)	8711 (10.98)	
Total cholesterol (n, %)			0.09
<200 mg/dL	10,546 (53.18)	45,225 (57.02)	
200–239 mg/dL	6414 (32.34)	24,911 (31.41)	
≥240 mg/dL	2870 (14.47)	9184 (11.58)	
CCI score (n, %)			0.10
0	11,085 (55.90)	49,249 (62.09)	
1	3341 (16.85)	12,084 (15.23)	
≥2	5404 (27.25)	17,987 (22.68)	
Asthma (n, %)	1898 (9.57)	6668 (8.41)	0.04

Abbreviation definitions: SD (standardized difference), SBP (systolic blood pressure), DBP (diastolic blood pressure), CCI (Charlson Comorbidity Index).

**Table 2:** Subgroup analyses of crude and overlap propensity score weighted hazard ratio (95% confidence interval) of gout for asthma.

Characteristics					Hazard Ratios for Asthma			
	N of Event /N of Total (%)	Follow-Up Duration (PY)	IR per 1000 (PY)	IRD (95% CI)	Crude †	*p*-Value	Adjusted †‡	*p*-Value
**Total participants**								
Gout	1898/19,830 (9.57)	151,948	12.50	1.60 (1.04 to 2.22)	1.14 (1.09 to 1.20)	<0.001 *	1.11 (1.05 to 1.17)	<0.001 *
Control	6668/79,320 (8.41)	613,929	10.90		1		1	
**Age < 60 years old**								
Gout	956/9752 (9.80)	88,335	10.80	1.64 (−1.17 to 3.70)	1.17 (1.09 to 1.26)	<0.001 *	1.14 (1.06 to 1.23)	0.001 *
Control	3273/39,008 (8.39)	357,449	9.16		1		1	
**Age ≥ 60 years old**								
Gout	942/10,078 (9.35)	63,613	14.80	1.60 (−2.19 to 4.70)	1.11 (1.04 to 1.20)	0.004 *	1.08 (1.00 to 1.16)	0.044 *
Control	3395/40,312 (8.42)	256,480	13.20		1		1	
**Male**								
Gout	1460/16,169 (9.03)	126,380	11.60	1.30 (−1.24 to 3.15)	1.12 (1.06 to 1.19)	<0.001 *	1.09 (1.03 to 1.16)	0.003 *
Control	5231/64,676 (8.09)	509,329	10.30		1		1	
**Female**								
Gout	438/3661 (11.96)	25,568	17.10	3.40 (−1.94 to 8.31)	1.23 (1.11 to 1.37)	<0.001 *	1.18 (1.06 to 1.32)	0.002 *
Control	1437/14,644 (9.81)	104,600	13.70		1		1	
**Low income**								
Gout	856/8256 (10.37)	60,685	14.10	2.60 (−0.84 to 5.67)	1.21 (1.12 to 1.31)	<0.001 *	1.17 (1.08 to 1.26)	<0.001 *
Control	2850/33,024 (8.63)	247,125	11.50		1		1	
**High income**								
Gout	1042/11,574 (9.00)	91,263	11.40	1.00 (−1.97 to 3.18)	1.09 (1.02 to 1.17)	0.012 *	1.06 (0.99 to 1.14)	0.096
Control	3818/46,296 (8.25)	366,804	10.40		1		1	
**Urban resident**								
Gout	777/8482 (9.16)	66,014	11.80	1.40 (−2.22 to 3.87)	1.13 (1.04 to 1.22)	0.004 *	1.08 (1.00 to 1.18)	0.050
Control	2768/33,928 (8.16)	265,760	10.40		1		1	
**Rural resident**								
Gout	1121/11,348 (9.88)	85,934	13.00	1.80 (−1.02 to 4.38)	1.16 (1.08 to 1.24)	<0.001 *	1.12 (1.05 to 1.20)	0.001 *
Control	3900/45,392 (8.59)	348,169	11.20		1		1	
**Underweight**								
Gout	18/258 (6.98)	2031	8.86	−1.54 (−18.15 to 15.13)	0.87 (0.53 to 1.42)	0.582	0.87 (0.53 to 1.43)	0.581
Control	150/1856 (8.08)	14,439	10.40		1		1	
**Normal weight**								
Gout	471/4953 (9.51)	37,937	12.40	1.80 (−1.13 to 6.69)	1.16 (1.05 to 1.28)	0.004 *	1.12 (1.02 to 1.24)	0.022 *
Control	2294/27,572 (8.32)	215,782	10.60		1		1	
**Overweight**								
Gout	507/5459 (9.29)	41,685	12.20	1.60 (−1.82 to 5.87)	1.14 (1.03 to 1.26)	0.010 *	1.12 (1.01 to 1.23)	0.029 *
Control	1849/22,510 (8.21)	174,584	10.60		1		1	
**Obese**								
Gout	902/9160 (9.85)	70,295	12.80	1.40 (−3.72 to 2.46)	1.13 (1.04 to 1.22)	0.002 *	1.11 (1.02 to 1.19)	0.011 *
Control	2375/27,382 (8.67)	209,124	11.40		1		1	
**Non-smoker**								
Gout	1103/10,563 (10.44)	81,566	13.50	1.70 (−3.72 to 1.83)	1.15 (1.08 to 1.23)	<0.001 *	0.88 (0.82 to 0.94)	<0.001 *
Control	3796/42,084 (9.02)	322,618	11.80		1		1	
**Past/current smoker**								
Gout	795/9267 (8.58)	70,382	11.30	1.44 (0.89 to 6.80)	1.13 (1.04 to 1.22)	0.003 *	0.92 (0.85 to 0.99)	0.035 *
Control	2872/37,236 (7.71)	291,311	9.86		1		1	
**Alcohol consumption < 1 time a week**								
Gout	1133/11,166 (10.15)	84,211	13.50	1.90 (−0.33 to 5.07)	1.15 (1.08 to 1.23)	<0.001 *	0.90 (0.84 to 0.96)	0.002 *
Control	4362/49,094 (8.88)	376,998	11.60		1		1	
**Alcohol consumption ≥ 1 time a week**								
Gout	765/8664 (8.83)	67,737	11.30	1.57 (−2.72 to 3.38)	1.16 (1.07 to 1.26)	0.001 *	0.88 (0.81 to 0.96)	0.003 *
Control	2306/30,226 (7.63)	236,931	9.73		1		1	
**SBP < 120 mmHg and DBP < 80 mmHg**								
Gout	1178/13,395 (8.79)	96,419	12.20	1.50 (1.38 to 6.52)	1.12 (1.05 to 1.20)	0.001 *	0.90 (0.84 to 0.96)	0.002 *
Control	4627/58,232 (7.95)	431,427	10.70		1		1	
**SBP ≥ 120 mmHg or DBP ≥ 80 mmHg**								
Gout	720/6435 (11.19)	55,529	13.00	1.80 (−2.89 to 3.57)	1.16 (1.06 to1.26)	0.001 *	0.89 (0.81 to 0.97)	0.006 *
Control	2041/21,088 (9.68)	182,502	11.20		1		1	
**Fasting blood glucose < 100 mg/dL**								
Gout	1183/11,172 (10.59)	89,776	13.20	1.90 (−0.79 to 4.32)	1.15 (1.08 to 1.23)	<0.001 *	0.87 (0.81 to 0.93)	<0.001 *
Control	4263/46,197 (9.23)	376,575	11.30		1		1	
**Fasting blood glucose ≥ 100 mg/dL**								
Gout	715/8658 (8.26)	62,172	11.50	1.40 (−3.59 to 3.01)	1.13 (1.04 to 1.23)	0.003 *	0.93 (0.85 to 1.01)	0.090
Control	2405/33,123 (7.26)	237,354	10.10		1		1	
**Total cholesterol < 200 mg/dL**								
Gout	957/10,546 (9.07)	77,783	12.30	1.60 (1.46 to 7.11)	1.13 (1.05 to 1.21)	0.001 *	0.91 (0.84 to 0.98)	0.008 *
Control	3701/45,225 (8.18)	344,446	10.70		1		1	
**Total cholesterol ≥ 200 mg/dL**								
Gout	941/9284 (10.14)	74,165	12.70	1.70 (−4.23 to 1.55)	1.15 (1.07 to 1.24)	<0.001 *	0.87 (0.81 to 0.94)	0.001 *
Control	2967/34,095 (8.70)	269,483	11.00		1		1	
**CCI score = 0**								
Gout	818/11085 (7.38)	82,647	9.90	0.86 (0.30 to 5.76)	1.08 (1.00 to 1.17)	0.041 *	0.89 (0.82 to 0.96)	0.003 *
Control	3395/49249 (6.89)	375,678	9.04		1		1	
**CCI score = 1**								
Gout	383/3341 (11.46)	25,367	15.10	1.10 (−6.04 to 4.09)	1.07 (0.96 to 1.20)	0.219	0.87 (0.78 to 0.98)	0.024 *
Control	1278/12,084 (10.58)	91,076	14.00		1		1	
**CCI scores ≥ 2**								
Gout	697/5404 (12.90)	43,934	15.90	2.30 (−2.94 to 4.52)	1.17 (1.07 to 1.27)	0.001 *	0.90 (0.82 to 0.98)	0.014 *
Control	1995/17,987 (11.09)	147,175	13.60		1		1	

Abbreviation definitions: IR (incidence rate), IRD (incidence rate difference), PY (person-year), CI (confidence interval), SBP (systolic blood pressure), DBP (diastolic blood pressure), and CCI (Charlson Comorbidity Index). * Stratified Cox proportional hazard regression model, significance at *p* < 0.05. † Models were stratified by age, sex, income, and region of residence. ‡ Models were adjusted for age, sex, income, region of residence, obesity, smoking, alcohol consumption, SBP, DBP, fasting blood glucose, total cholesterol, and CCI scores.

**Table 3:** Subgroup analyses of crude and overlap propensity score weighted hazard ratio (95% confidence interval) of gout for asthma exacerbation.

Characteristics					Hazard Ratios for Asthma			
	N of Event /N of Total (%)	Follow-Up Duration (PY)	IR per 1000 (PY)	IRD (95% CI)	Crude †	*p*-Value	Adjusted †‡	*p*-Value
**Total partic** **i** **pants**								
Gout	310/1898 (16.33)	21,223	14.60	0.80 (−1.03 to 2.58)	1.05 (0.93 to 1.19)	0.437	0.99 (0.87 to 1.12)	0.842
Control	1027/6668 (15.40)	74,256	13.80		1		1	
**Age < 60 years old**								
Gout	135/956 (14.12)	11,329	11.90	1.90 (−0.23 to 4.05)	1.18 (0.97 to 1.44)	0.090	1.06 (0.87 to 1.30)	0.547
Control	390/3273 (11.92)	38,976	10.00		1		1	
**Age ≥ 60 years old**								
Gout	175/942 (18.58)	9894	17.70	−0.40 (−3.36 to 2.62)	0.97 (0.82 to 1.15)	0.761	0.94 (0.79 to 1.11)	0.437
Control	637/3395 (18.76)	35,280	18.10		1		1	
**Male**								
Gout	226/1460 (15.48)	16,442	13.70	0.00 (−2.03 to 2.03)	1.00 (0.86 to 1.16)	0.970	0.96 (0.83 to 1.12)	0.599
Control	801/5231 (15.31)	58,266	13.70		1		1	
**Female**								
Gout	84/438 (19.18)	4781	17.60	3.50 (−0.51 to 7.38)	1.23 (0.96 to 1.58)	0.108	1.08 (0.84 to 1.40)	0.531
Control	226/1437 (15.73)	15,990	14.10		1		1	
**Low income**								
Gout	157/856 (18.34)	9384	16.70	1.20 (−1.67 to 4.13)	1.08 (0.90 to 1.29)	0.403	1.04 (0.87 to 1.25)	0.656
Control	483/2850 (16.95)	31,153	15.50		1		1	
**High income**								
Gout	153/1042 (14.68)	11,839	12.90	0.30 (−1.99 to 2.59)	1.02 (0.85 to 1.21)	0.872	0.93 (0.78 to 1.12)	0.448
Control	544/3818 (14.25)	43,103	12.60		1		1	
**Urban resident**								
Gout	124/777 (15.96)	8621	14.40	2.00 (−0.68 to 4.72)	1.15 (0.94 to 1.40)	0.184	1.10 (0.90 to 1.35)	0.356
Control	385/2768 (13.91)	31,147	12.40		1		1	
**Rural resident**								
Gout	186/1121 (16.59)	12,602	14.80	−0.10 (−2.55 to 2.29)	0.99 (0.84 to 1.17)	0.929	0.93 (0.78 to 1.09)	0.357
Control	642/3900 (16.46)	43,109	14.90		1		1	
**Underweight**								
Gout	7/18 (38.89)	146	47.90	22.30 (−32.46 to 76.74)	1.62 (0.72 to 3.62)	0.243	2.85 (1.17 to 6.97)	0.022 *
Control	38/150 (25.33)	1483	25.60		1		1	
**Normal weight**								
Gout	91/471 (19.32)	5039	18.10	4.50 (−4.70 to 13.40)	1.29 (1.03 to 1.63)	0.029 *	1.18 (0.93 to 1.49)	0.168
Control	350/2294 (15.26)	25,741	13.60		1		1	
**Overweight**								
Gout	79/507 (15.58)	5666	13.90	0.90 (−8.79 to 8.79)	1.07 (0.83 to 1.37)	0.620	0.95 (0.74 to 1.23)	0.719
Control	268/1849 (14.49)	20,664	13.00		1		1	
**Obese**								
Gout	133/902 (14.75)	10,372	12.80	−1.30 (−9.89 to 3.68)	0.92 (0.76 to 1.12)	0.421	0.88 (0.72 to 1.07)	0.205
Control	371/2375 (15.62)	26,368	14.10		1		1	
**Non-smoker**								
Gout	191/1103 (17.32)	12,368	15.40	2.60 (0.28 to 4.92)	1.20 (1.01 to 1.41)	0.034 *	1.10 (0.94 to 1.31)	0.241
Control	550/3796 (14.49)	42,832	12.80		1		1	
**Past/current smoker**								
Gout	119/795 (14.97)	8855	13.40	−1.80 (−4.61 to 1.13)	0.88 (0.72 to 1.08)	0.222	0.85 (0.69 to 1.04)	0.118
Control	477/2872 (16.61)	31,424	15.20		1		1	
**Alcohol consumption <1 time a week**								
Gout	193/1133 (17.03)	12,615	15.30	1.90 (−0.41 to 4.19)	1.13 (0.96 to 1.33)	0.136	1.05 (0.89 to 1.24)	0.546
Control	656/4362 (15.04)	48,929	13.40		1		1	
**Alcohol consumption ≥ 1 time a week**								
Gout	117/765 (15.29)	8608	13.60	−1.00 (−3.99 to 1.88)	0.93 (0.76 to 1.15)	0.504	0.89 (0.72 to 1.09)	0.263
Control	371/2306 (16.09)	25,327	14.60		1		1	
**SBP < 120 mmHg and DBP < 80 mmHg**								
Gout	54/358 (15.08)	96,419	12.20	1.20 (−2.68 to 5.07)	1.09 (0.81 to 1.47)	0.567	1.03 (0.76 to 1.40)	0.830
Control	217/1573 (13.80)	431,427	10.70		1		1	
**SBP ≥ 120 mmHg or DBP ≥ 80 mmHg**								
Gout	256/1540 (16.62)	17,256	14.80	0.50 (−1.48 to 2.61)	1.03 (0.90 to 1.19)	0.638	0.98 (0.85 to 1.13)	0.740
Control	810/5095 (15.90)	56,776	14.30		1		1	
**Fasting blood glucose < 100 mg/dL**								
Gout	196/1183 (16.57)	13,391	14.60	0.70 (−1.54 to 3.01)	1.05 (0.90 to 1.24)	0.521	0.99 (0.84 to 1.17)	0.922
Control	664/4263 (15.58)	47,765	13.90		1		1	
**Fasting blood glucose ≥ 100 mg/dL**								
Gout	114/715 (15.94)	7832	14.60	0.90 (−2.12 to 3.82)	1.05 (0.85 to 1.29)	0.662	0.98 (0.80 to 1.22)	0.878
Control	363/2405 (15.09)	26,491	13.70		1		1	
**Total cholesterol < 200 mg/dL**								
Gout	172/957 (17.97)	10,486	16.40	2.20 (−0.38 to 4.81)	1.14 (0.97 to 1.36)	0.121	1.09 (0.91 to 1.29)	0.346
Control	580/3701 (15.67)	40,891	14.20		1		1	
**Total cholesterol ≥ 200 mg/dL**								
Gout	138/941 (14.67)	10,737	12.90	−0.50 (−3.05 to 1.96)	0.96 (0.79 to 1.16)	0.672	0.89 (0.73 to 1.08)	0.222
Control	447/2967 (15.07)	33,365	13.40		1		1	
**CCI score = 0**								
Gout	65/818 (7.95)	9766	6.66	0.34 (−6.79 to 6.02)	1.05 (0.80 to 1.38)	0.724	1.03 (0.78 to 1.36)	0.814
Control	255/3395 (7.51)	40,347	6.32		1		1	
**CCI score = 1**								
Gout	73/383 (19.06)	4149	17.60	−0.20 (−11.35 to 9.82)	0.98 (0.75 to 1.27)	0.880	0.96 (0.74 to 1.26)	0.787
Control	244/1278 (19.09)	13,731	17.80		1		1	
**CCI scores ≥ 2**								
Gout	172/697 (24.68)	7308	23.50	−2.70 (−11.87 to 4.88)	0.90 (0.76 to 1.07)	0.253	0.92 (0.77 to 1.10)	0.352
Control	528/1995 (26.47)	20,178	26.20		1		1	

Abbreviation definitions: IR (incidence rate), IRD (incidence rate difference), PY (person-year), CI (confidence interval), SBP (systolic blood pressure), DBP (diastolic blood pressure), and CCI (Charlson Comorbidity Index). * Stratified Cox proportional hazard regression model, significance at *p* < 0.05. † Models were stratified by age, sex, income, and region of residence. ‡ Models were adjusted for age, sex, income, region of residence, obesity, smoking, alcohol consumption, SBP, DBP, fasting blood glucose, total cholesterol, and CCI scores.

## Data Availability

These data have certain limitations in terms of availability. They were obtained from the Health Insurance Review and Assessment Service (HIRA) of Korea and can be accessed via https://opendata.hira.or.kr (accessed on 20 September 2023) with authorization from HIRA.
